# Complete genome sequence of a species of the genus *Agarivorans* from the Sea of Japan

**DOI:** 10.1128/mra.01234-23

**Published:** 2024-02-15

**Authors:** Yuka Kawase, Masa-aki Yoshida

**Affiliations:** 1 Oki Marine Biological Station, Faculty of Life and Environmental Sciences, Shimane University, Matsue, Shiman, Japan; University of Southern California, Los Angeles, California, USA

**Keywords:** seaweed, marine ecosystems, agarase

## Abstract

A strain of *Agarivorans* sp., named OAG1, predicted to be capable of degrading agar, was isolated from the Sea of Japan. Its 4.7 Mbp genome with 4,224 predicted protein-coding genes offers insight into the varied genes responsible for agar degradation, as observed in the comparative genomic study of *Agarivorans* species.

## ANNOUNCEMENT

To date, four species under the genus *Agarivorans* have been identified: *Agarivorans albus*, *Agarivorans gilvus*, *Agarivorans aestuarii*, and *Agarivorans litoreus* (1–4). These are Gram-negative, aerobic bacteria known for their agar-degrading capacity, a feature that potentially influences the carbon cycle in marine ecosystems (1–4). We isolated *Agarivorans* sp. strain OAG1 from a seawater-immersed plastic surface and presented the complete genome sequence.

Microbial communities were obtained from a plastic film submerged in the Sea of Japan (36.178856 N, 133.280109 E) for several weeks. This particular strain was isolated from a poly(3-hydroxybutyrate-*co*-3-hydroxyhexanoate) film immersed for 60 days. Bacterial biofilms on the film were collected by vigorous vortexing. Serial limiting dilutions of 10× each were made, and the diluted inoculum (20 µL) was transferred to marine broth (200 µL, Difco, NJ, USA) for 1 week at 25°C. Subsequently, the 10^7^–10^8^ culture was transferred to marine agar (Difco) and strains culminating in isolated colonies were collected. The colonies were collected and subsequently used for glycerol stock (10% glycerol/seawater) and recultured in marine broth for 5 days for the following usage. Genomic DNA was extracted using the NucleoSpin Microbial DNA kit (TakaraBio, Japan). Genomic sequencing for both long-read and short-read sequencing was performed using the same DNA with the protocol proposed by Genome-Lead Inc. (5). One microgram of unfragmented gDNA was size-selected using Short Read Eliminator XS (Circulomics, CA, USA), and the obtained 600 ng was used for library construction by a ligation sequencing kit [SQK-LSK109; Oxford Nanopore Technologies (ONT), UK]. Long-read sequencing using GridION X5 with a flowcell R9.4.1 (ONT) produced 100,141 reads (1.09 Gb) post-processing with Guppy v.4.0.11 (high-accuracy basecalling), with the estimated *N*50 value of 14.4 kbp. After quality trimming (average Phred quality value > 10) using NanoFilt v.2.7.1 (6), 84,822 reads (937 Mb) were retained. For short-read sequencing, 400 ng of gDNA was processed using the MGIEasy FS PCR-Free DNA Library Prep Set (MGI, China). Paired-end (2  ×  150 bp) reads were obtained using a DNBSEQ (MGI, China). The raw sequencing data (8,612,228 reads) were processed using the fastp v.0.20.1 (qualified Phred quality score: 30) (7) to trim adapters and low-quality data, yielding 6,662,136 reads.

The Unicycler v.0.4.8 pipeline facilitated the complete genome assembly (8). Polished, circular assemblies were inspected using Bandage v.0.8.1 (9). Contaminating DNA checks were conducted using BlobTools (10), and the genome was polished three times using Pilon v.1.24 (11). The final assembly was 4,773,720 bp long with 44.3% GC content. Genome annotation utilized DFAST v.1.6.0 (12) and eggNOG-mapper v.2 (13), identifying 4,224 protein-coding sequences, 22 rRNA, and 92 tRNA genes. Average nucleotide identity (ANI) values were determined using the ANI Calculator (14). Default parameters were used for all software unless otherwise specified.

A webBLAST search of the 16S rDNA sequence of OAG1 revealed a 99.29% similarity to *Agarivorans albus* JCM21469 (as of October 2023). ANI analysis also showed the highest sequence similarity (97.13%) to *A. albus* JCM21469 (NCBI accession number: NZ_AP023032.1). A large inversion of 3,490,405 bp was observed when compared to *A. albus* JCM21469, suggesting that it occurred in the OAG1 lineage.

## Data Availability

An assembled chromosome sequence was deposited in DDBJ under the accession number AP028990.1. Raw ONT and DNBSEQ reads are available in the DDBJ Sequence Read Archive (DRA) under BioProject accession number PRJDB14979 and DRA accession numbers DRX492827 and DRX492828.
